# Supplementary material for: Evaluating the inter-species transmission risk of amyloid beta peptide aggregates via ingestion
Source: Alzheimers Res Ther. 2024 Jun 7;16:123. doi: 10.1186/s13195-024-01487-8 (PMC11157902; doi:10.1186/s13195-024-01487-8)
Supplement: Supplementary file 2 — Supplementary Material 2. Full mass spectrometry methods. Contains detailed descriptions of the mass spectrometry methods, false discovery rate graphs for analysis, and peptide sequences used to query the database. [file 13195_2024_1487_MOESM2_ESM.pdf]

Supplementary information for Raine *et al.* "Evaluating the transmission risk of amyloid beta peptide via ingestion"

S2 - Full mass spectrometry methods

LCMS Data Acquisition was performed under the following parameters. Eksigent NanoLC-Ultra with trap column; Trajan ProteoCol C18P 3 µm 120 Å, 300 µm x 10 mm and analytical column; Thermo Scientific Acclaim PepMap100 C18 3 µm 100 Å, 75 µm x 250 mm. The following solvent ratios were used: Solvent A: 2% acetonitrile, 0.1% formic acid and Solvent B: 98% acetonitrile, 0.1% formic acid.

Table S2.1. LC solvent scheme.

Time	Flow rate (nL/min)	% A	% B
0	300	95	5
20	300	88	12
60	300	70	30
62	300	10	90
69	300	10	90
72	300	95	5
85	300	95	5

SCIEX TripleTOF 5600 with the ionization parameters: Nebulizer gas (GS1) 12 units, Curtain gas 30 units, IonSpray voltage floating 2300 V, and Interface heater temperature 150 °C. MS – IDA (information-dependent acquisition) parameters: Mass range 350 – 1250 m/z, accumulation time 250 msec, maximum 20 precursors selected for fragmentation, charge state 2-4, intensity > 125 cps, dynamic exclusion for 15 sec. MSMS parameters: mass range 100 – 1800 m/z, accumulation time: 100 msec, mode High sensitivity, rolling collision energy enabled.

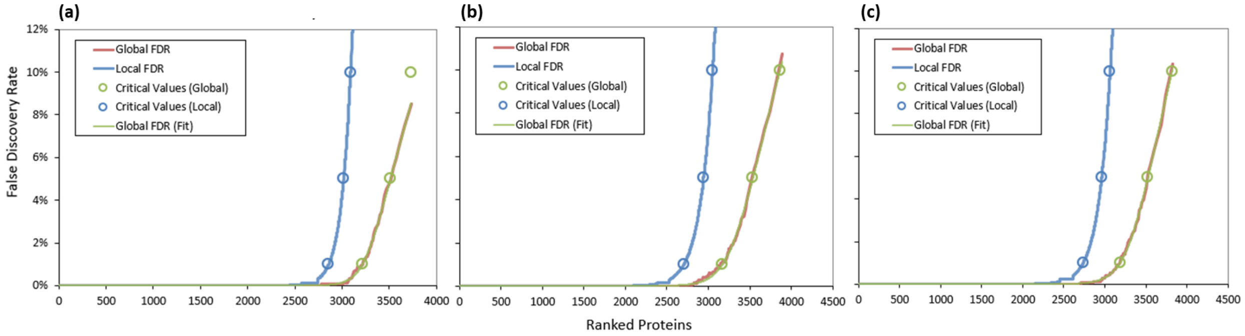

Figure S2.1. Protein false discovery rates for (**a**) Aβ expressing *D. melanogaster* larvae, (**b**) Aβ fed *D. rerio* brain tissue and (**c**) tdTomato fed *D.rerio* brain tissue. Analysis performed in ProteinPilot software 5.03.

Table S2.2. Query sequences used to search the peptide MS data. The regions highlighted indicate peptide coverage. **Green:** High confidence > 95%, **Orange:** Medium confidence 95% - 20%, **Red:** Low confidence, < 20%. Analysis performed in ProteinPilot software 5.03.

Query label	Query amino acid sequence and peptide coverage
Aβ_{42} peptide	MDAEFRHDSGYEVHHQKLVFFAEDVGSNKGAIIGLMVGGVVIA
mCherry	MVSKGEEDNMAIIKEFMRFKVHMEGSVNGHEFEIEGEGEGRPYEGTQTAKLKVTKG<br>GPLPFAWDILSPQFMYGSKAYVKHPADIPDYLKLSFPEGFKWERVMNFEDGGVVTVT<br>QDSSLQDGEFIYKVKLRGTNFPSDGPVMQKKTMGWEASSERMYPEDGALKGEIKQR<br>LKLKDGGHYDAEVKTTYKAKKPVQLPGAYNVNIKLDITSHNEDYTIVEQYERAEGR<br>HSTGGMDELYK
CRY2	MKMDKKTIVWFRRDLRIEDNPALAAAAHEGSVFPVFIWCPEEEGQFYPGRASRWW<br>MKQSLAHLSQSLKALGSDLTLIKTHNTISAILDCIRVTGATKVVFNHLYDPVSLVRDH<br>TVKEKLVERGISVQSYNGDLLYEPWEIYCEKGKPFTSFNSYWKKCLDMSIESVMLPPP<br>WRLMPITAAAEAIWACSIEELGLENEAEKPSNALLTRAWSPGWSNADKLLNEFIEKQ<br>LIDYAKNSKKVVGNSTSLLSPYLHFGEISVRHVFQCARMKQIIWARDKNSEGEESADL<br>FLRGIGLREYSRYICFNFPFTHEQSLLSHLRFFPWDADVDKFKAWRQGRTGYPLVDAG<br>MRELWATGWMHNRIRVIVSSFAVKFLLLPWKWGMKYFWDTLLDADLECDILGWQY<br>ISGSIPDGHELDRLDNPALQGAKYDPEGEYIRQWLPELARLPTEWIHHPWDAPLTVL<br>KASGVELGTNYAKPIVDIDTARELLAKAISRTREAQIMIGAAARDPP

Table S2.3. Full detection of target proteins via MS in assorted tissues from figure 2. ProtScore >1.64 = < 1 % Local false discovery rate. ProtScore >0.47 = <1 % Global false discovery rate. '-' indicates no peptide evidence detected for analysis. Peptide confidence >95%. Analysis performed in ProteinPilot software 5.03.

Subject	Aβ fed Zebrafish gut #1			Aβ fed Zebrafish gut #2			Aβ fed Zebrafish gut #3			Control fed Zebrafish gut #1, #2, #3		
Query sequence	Aβ peptide	mCherry	CRY2	Aβ peptide	mCherry	CRY2	Aβ peptide	mCherry	CRY2	Aβ peptide	mCherry	CRY2
Peptides	0	0	0	0	18	0	0	10	0	0	0	0
Cover (%)	0	0	0	0	57.87	0	0	26.81	0	0	0	0
Unused ProtScore	-	-	-	-	21.21	-	-	8.25	-	-	-	-
Total ProtScore	-	-	-	-	21.29	-	-	8.28	-	-	-	-

Subject	Aβ expressing larvae #1			Aβ expressing larvae #2			Control expressing larvae #1			Control expressing larvae #2		
Query sequence	Aβ peptide	mCherry	CRY2	Aβ peptide	mCherry	CRY2	Aβ peptide	mCherry	CRY2	Aβ peptide	mCherry	CRY2
Peptides	1	13	20	1	13	20	0	7	0	0	6	0
Cover (%)	27.91	59.15	42.15	27.91	58.72	44.14	0	26.66	0	0	27.23	0
Unused ProtScore	2	23.57	38.74	2	23.64	37.98	-	4.04	-	-	6.02	-
Total ProtScore	2	23.61	38.75	2	23.66	38.01	-	4.04	-	-	6.03	-
